# Heat shock protein 70 kDa (HSP70) is involved in the maintenance of pig sperm function throughout liquid storage at 17 °C

**DOI:** 10.1038/s41598-024-64488-5

**Published:** 2024-06-11

**Authors:** Ferran Garriga, Carolina Maside, Lorena Padilla, Sandra Recuero, Joan E. Rodríguez-Gil, Marc Yeste

**Affiliations:** 1 Biotechnology of Animal and Human Reproduction (TechnoSperm), Institute of Food and Agricultural Technology, University of Girona, 17003 Girona, Spain; 2 Unit of Cell Biology, Department of Biology, Faculty of Sciences, University of Girona, 17003 Girona, Spain; 3 Unit of Animal Reproduction, Department of Animal Medicine and Surgery, Faculty of Veterinary Medicine, Autonomous University of Barcelona, 08193 Bellaterra, Cerdanyola del Vallès, Barcelona Spain; 4 Catalan Institution for Research and Advanced Studies (ICREA), 08010 Barcelona, Spain

**Keywords:** HSP70, YM-1, Sperm, Liquid storage, Flow cytometry, Pig, Animal biotechnology, Biological techniques, Cell biology, Developmental biology, Zoology

## Abstract

At present, liquid storage is the most efficient method for pig semen preservation. This approach relies upon reducing sperm metabolism, allowing for the maintenance of cell lifespan. In this context, the study of proteins that could protect sperm during liquid storage is of high relevance. The 70 kDa Heat Shock Protein (HSP70) is an anti-apoptotic protein that has been reported to be relevant to sperm survival. Thus, we explored the role of HSP70 during prolonged storage of pig semen at 17 °C. Six semen pools were incubated with YM-1 (0, 0.05, 0.1 and 0.2 μM), an HSP70 inhibitor, and stored at 17 °C for 21 days. On days 0, 4, 10, 14 and 21, sperm quality and function were evaluated through flow cytometry and Computer-Assisted Sperm Analysis (CASA), and HSP70 activity and chromatin condensation were also determined. While inhibition of HSP70 increased progressive motility, Ca^2+^ and Reactive Oxygen Species (ROS) levels, and mitochondrial activity during the first 10 days of storage, it had a detrimental effect on sperm motility after 14 and 21 days. In spite of this, sperm viability was not altered. We can conclude that HSP70 contributes to the liquid storage of pig semen because it keeps mitochondrial activity low, which is needed for the maintenance of sperm function.

## Introduction

Artificial insemination (AI) is the most used reproductive technique for pig breeding, as it displays high efficiency and productivity^1^. Indeed, it offers many advantages—including genetic selection—and allows for improving the sanitary control of semen^2,3^. Boars are collected and seminal doses are prepared in a different place to where they are used for AI; thus, they need to be preserved and transported to AI centers. Mainly, there are two methods for sperm preservation: liquid storage and cryopreservation. In pigs, almost 99% of the AIs are conducted using semen stored at 15–20 °C^4^. This preference is based on the greater reproductive performance of liquid preserved semen compared to frozen-thawed sperm, which has cryoinjuries associated^4,5^. Moreover, AI with liquid stored semen is easier and cheaper than the one employing cryopreserved sperm^6^. In spite of this, pig sperm are very sensitive to low temperatures, so liquid storage needs to be performed at higher temperatures (15–20 °C) than those employed in other mammalian species (4–5 °C).

Apart from potential microbial contamination, which is greater at 15–20 °C than at 4–5 °C, the main inconvenience of storing sperm at 15–20 °C is that these temperatures only reduce cell metabolism partially, so that apoptotic-like changes may occur, thus becoming a limitation for sperm lifespan^4^. Sperm ageing is, therefore, one of the main concerns for liquid preservation. Several studies reported that liquid storage of semen from different farm animals compromises sperm quality in the mid-term, especially by altering Ca^2+^ homeostasis, membrane fluidity and sperm motility^7,8^. This loss of sperm quality during liquid storage seems to be related to the induction of apoptotic-like changes in the male gamete. As described by Kumaresan et al.^9^, storage of pig semen at 18 °C induces lipid peroxidation, increases plasma membrane fluidity and reduces mitochondrial membrane potential (MMP), even during the first 4 days of storage. In pig sperm, the induction of these alterations has been described to be tightly related to mitochondrial function^10^. Besides, in human sperm, apoptotic-like changes are suggested to be induced by Ca^2+^ overload, which triggers the apoptotic cascade^11^.

The study of proteins that prevent apoptotic-like changes is of high interest for the preservation of semen. The heat shock proteins (HSPs) family includes both a group of chaperones that are induced by stress conditions to promote cell survival, and others that are expressed constitutively and are known to play a role in regulating apoptosis^12,13^. Heat shock protein 70-kDa (HSP70), also known as HSPA1A, has an anti-apoptotic role^14^. The presence of HSP70 in mature pig sperm was studied by Spinaci et al.^15^, who reported that this protein is localized in the equatorial segment of fresh sperm and relocates during capacitation and the acrosome reaction. Moreover, it has been observed that low levels of this protein are associated with reduced sperm quality in pigs^16^, and that the resilience acquired by sperm during the holding time before cryopreservation is related to its serine-phosphorylation^17^. Different agents have been used to investigate the importance of HSP70 for cell function. One of these drugs is YM-1, a cell-permeable analog of MKT-077 that acts as an allosteric HSP70 inhibitor and promotes the closed status of HSP70 by interacting with its nucleotide binding domain^18^. In humans, YM-1 leads to death in HeLa cells and promotes proteostasis in neural cells^19,20^. Yet, neither the repercussion of inhibiting HSP70 with YM-1 on mammalian sperm, nor how this could affect their preservation at 15–20 °C has been interrogated.

Considering all the exposed above, we sought to determine the role of HSP70 on sperm quality and functionality during liquid storage of pig semen at 17 °C for 21 days through its inhibition with YM-1.

## Results

### YM-1 inhibits HSP70 activity in sperm during liquid storage at 17 °C

**Figure 1:**
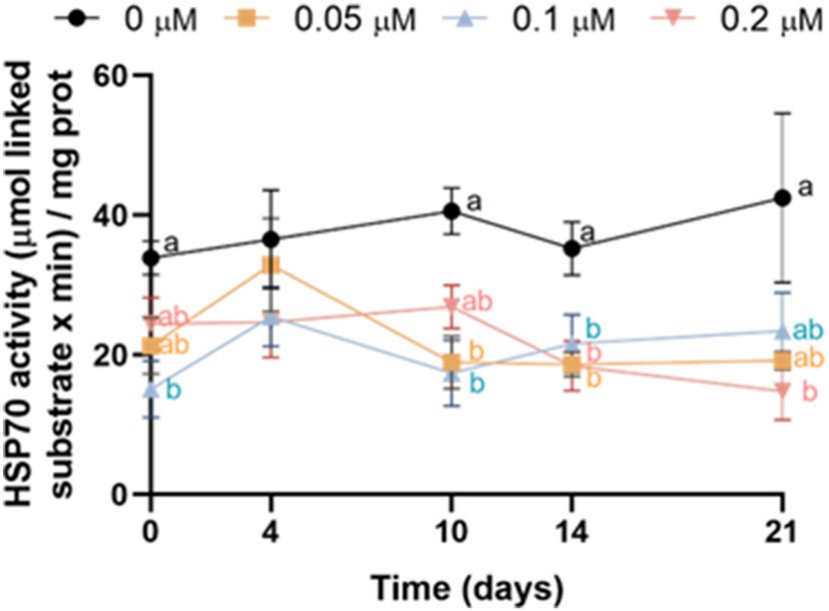
Inhibition of HSP70 with YM-1 led to a decrease in HSP70 activity during liquid storage of pig semen at 17 °C. Different letters indicate significant differences (*P* ≤ 0.05) between experimental groups (0, 0.05, 0.1 and 0.2 μM of YM-1) on a given time point (0, 4, 10, 14 and 21 days). Results are expressed as the mean ± SEM (N = 6).

Our results showed that incubation of pig sperm with YM-1 decreased HSP70 activity, especially when evaluated after a long period of storage (Fig. 1). An immediate effect was observed after the addition of 0.1 YM-1 on day 0, with a significant decrease of HSP70 activity. Moreover, HSP70 activity was significantly lower when sperm were treated with 0.05 µM and 0.1 µM of YM-1 for 10 days, and with 0.05 µM and 0.2 µM of YM-1 for 14 days. Similarly, a trend in the case of the other treatments was detected for 0.1 µM of YM-1 on day 14 (*P* = 0.068), and for 0.2 µM of YM-1 on day 21 (*P* = 0.065).

### Inhibition of HSP70 activity does not compromise the integrity of plasma and acrosome membranes

**Figure 2:**
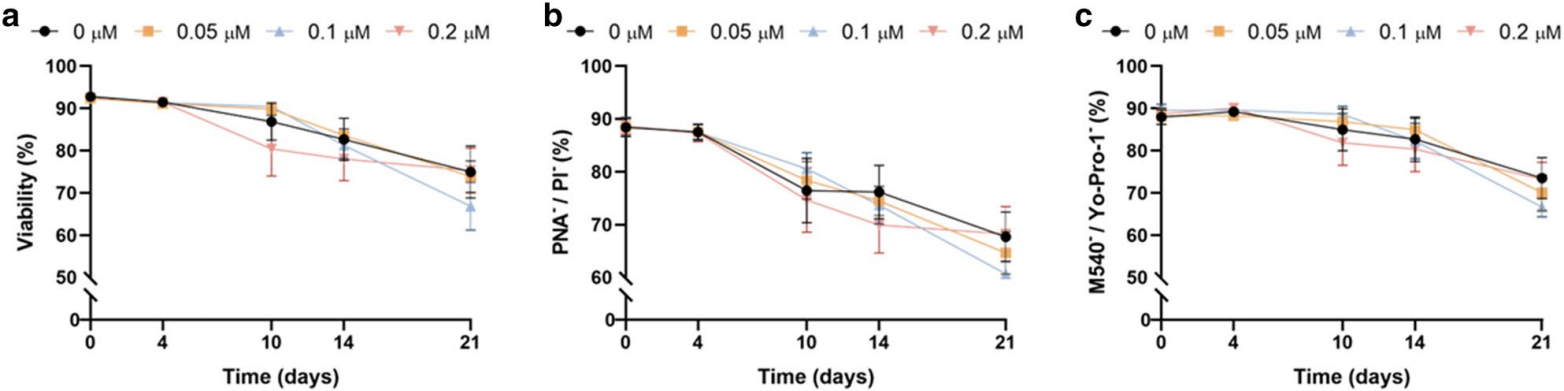
Inhibition of HSP70 activity had no effect on plasma and acrosome integrity during liquid storage of pig semen at 17 °C. (**a**) Plasma membrane integrity (SYBR-14/PI); (**b**) acrosome integrity (PNA/PI); and (**c**) membrane lipid disorder (M540/Yo-Pro-1). Results are expressed as the mean ± SEM (N = 6).

The integrity of plasma (SYBR-14/PI) and acrosome membranes (PNA-FITC/PI), as well as membrane lipid disorder (M540/Yo-Pro-1) of sperm, were evaluated through flow cytometry, and are represented in Fig. 2a–c, respectively. Neither the integrity of plasma and acrosome membranes, nor lipid disorder were impaired when HSP70 activity was inhibited with YM-1.

### Inhibition of HSP70 activity alters mitochondrial activity, and intracellular levels of ROS and Ca^2+^

**Figure 3:**
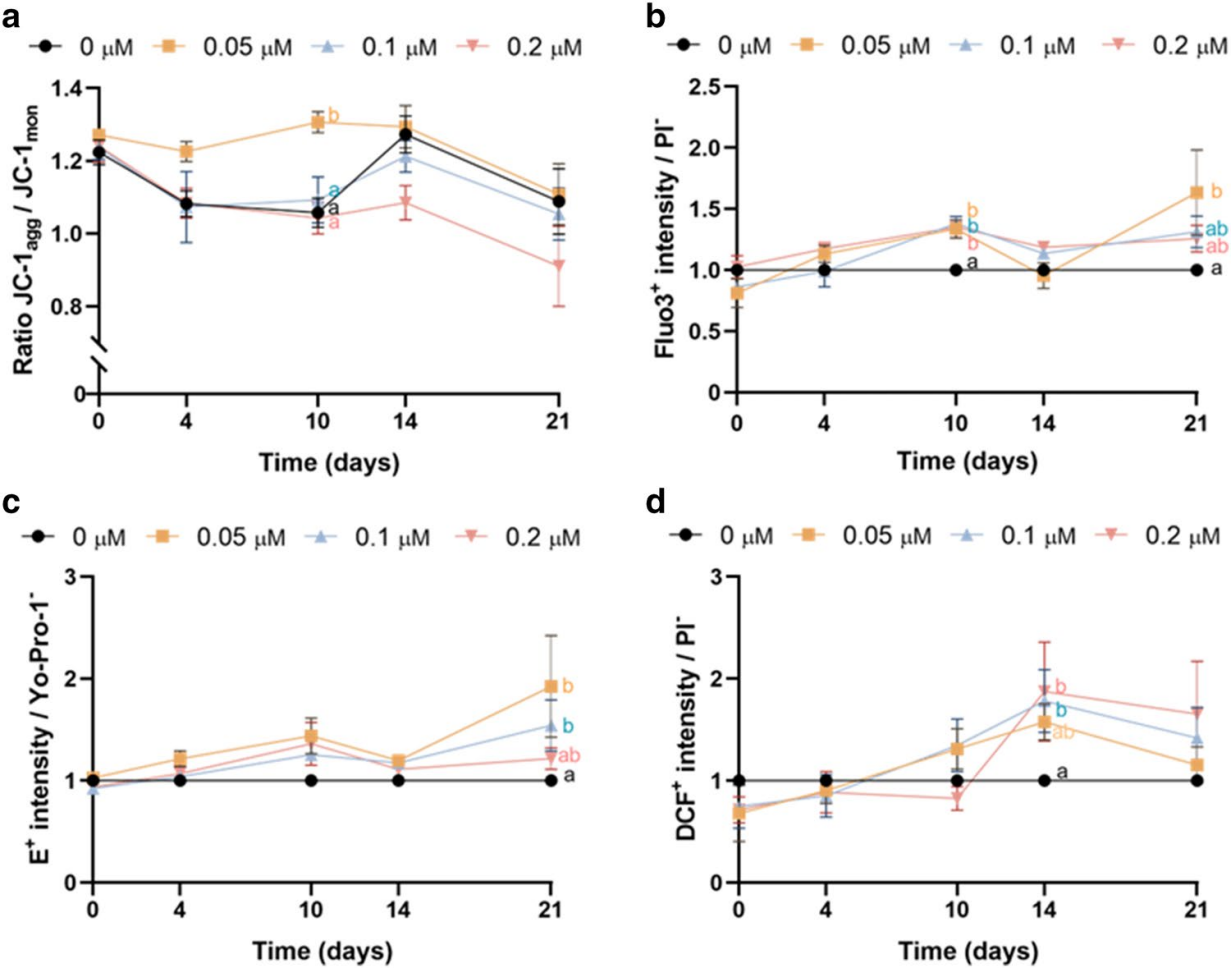
Effects of HSP70 activity inhibition during liquid storage of pig semen at 17 °C on (**a**) ratio of intensity between JC-1_agg_ and JC-1_mon_; (**b**) intracellular Ca^2+^ levels (Fluo3^+^) in viable sperm (PI^−^); (**c**) intracellular superoxide levels (E^+^) in viable sperm (Yo-Pro-1^−^); and (**d**) intracellular total ROS levels (DCF^+^) in viable sperm (PI^−^). Different letters indicate significant differences (*P* ≤ 0.05) between experimental groups (0, 0.05, 0.1 and 0.2 μM of YM-1) on a given time point (0, 4, 10, 14 and 21 days). Results are expressed as the mean ± SEM (N = 6).

Mitochondrial membrane potential (JC-1), and intracellular levels of superoxides (HE/Yo-Pro-1), total ROS (H_2_DCFDA/PI) and Ca^2+^ (Fluo3/PI) were also evaluated through flow cytometry. Inhibition of HSP70 activity with the lowest concentration of YM-1 led to a significant increase in the ratio of JC-1_agg_/JC-1_mon_ after 10 days of storage (Fig. 3a). Moreover, this inhibition also increased Ca^2+^ levels (Fluo3^+^ intensity) on day 10 in all treatments (Fig. 3b). This effect was also observed when sperm were treated with the lowest concentration of YM-1 for 21 days. Regarding the effects of HSP70 inhibition on intracellular ROS levels, incubation with the two lowest concentrations of YM-1 raised the levels of superoxides (measured as E^+^ intensity) after 21 days of storage (Fig. 3c). Inhibition of HSP70 activity with 0.1 and 0.2 µM of YM-1 also increased total ROS levels, as determined by DCF^+^ intensity, on day 14 (Fig. 3d).

### Inhibiting HSP70 activity increases progressive, but not total motility over the first days of semen storage

**Figure 4:**
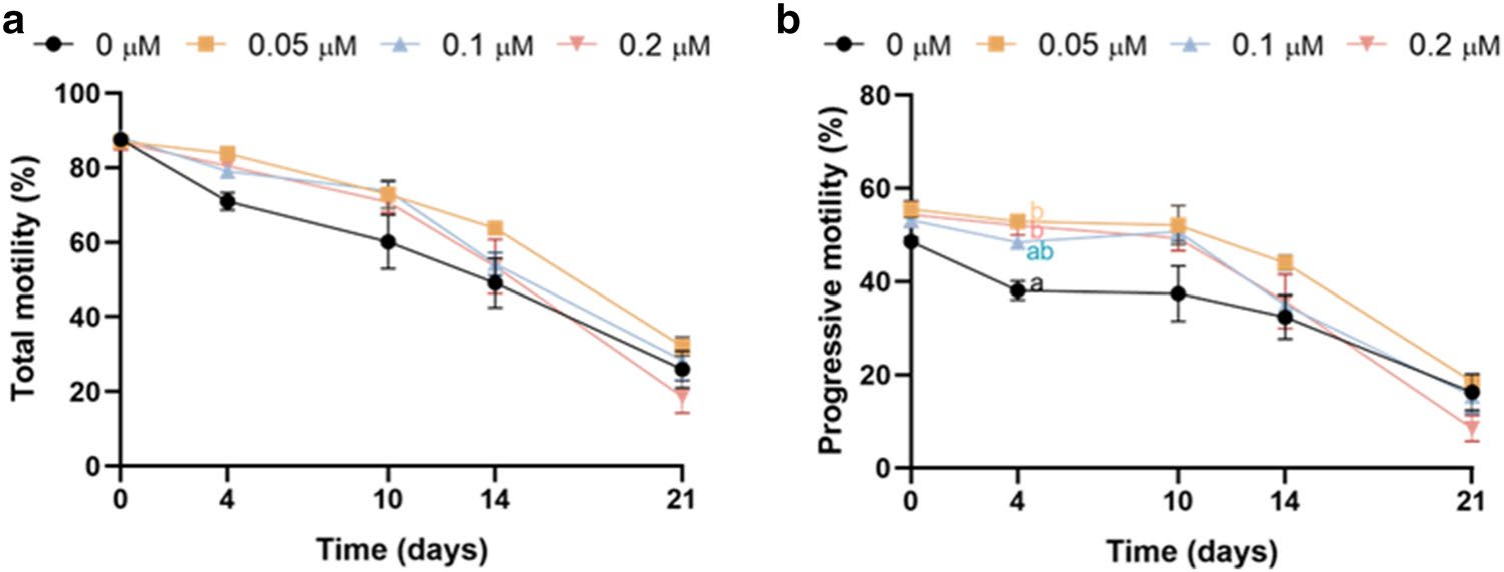
Inhibition of HSP70 activity with YM-1 on sperm motility during liquid storage of pig semen at 17 °C. While the percentage of progressively motile sperm increased on days 4 and 10 of storage (**a**); no effect on the percentage of total motility was observed (**b**). Different letters indicate significant differences (*P* ≤ 0.05) between experimental groups (0, 0.05, 0.1 and 0.2 μM of YM-1) on a given time point (0, 4, 10, 14 and 21 days). Results are expressed as the mean ± SEM (N = 6).

Inhibition of HSP70 activity with the lowest concentration of YM-1 significantly increased progressive motility after 4 days of storage; a similar trend (*P* = 0.087) was observed at a concentration of 0.2 µM (Fig. 4a). On day 10, this tendency (*P* = 0.061) was also seen in the treatment with the lowest concentration of the inhibitor. In contrast, no significant differences in total motility were found between treatments (Fig. 4b).

### Inhibition of HSP70 activity alters the distribution of motile sperm subpopulations

The PCA analysis of sperm kinematic parameters showed the existence of two principal components, which explained up to 79.9% of the variance. These components were tightly related to VCL, VSL, VAP, LIN, STR, WOB, ALH and BCF, as detailed in Table 1. Subsequently, a cluster analysis was performed by using these components. A total of three sperm subpopulations, exhibiting different kinematics, were obtained. Table 2 describes the kinematic characteristics of the sperm belonging to each subpopulation. Subpopulation 1 (SP1) comprised the slowest non-linear sperm and represented 33.41% of total motile sperm. Subpopulation 2 (SP2) was characterized by fast sperm but with low linearity. This population included 34.21% of motile sperm. The other 29.38% of motile sperm belonged to Subpopulation 3 (SP3), which consisted of sperm with intermediate velocity and high linearity.

**Table 1:** Resulting PCA components based on kinematic characteristics evaluated by CASA. The factor loading (a_ij_^2^) represents the correlation between a given kinematic parameter and the corresponding principal component.

Principal component	Variance (%)	Parameter	a_ij_^2^
Component 1	46.90	VCL	0.97
		VAP	0.86
		ALH	0.87
		BCF	0.74
Component 2	32.96	VSL	0.78
		LIN	0.98
		STR	0.84
		WOB	0.75

VCL, Curvilinear velocity; VAP, Average path velocity; ALH, Amplitude of lateral head displacement; BCF, Beat-cross frequency; VSL, Straight-line velocity; LIN, Linearity; STR, Straightness; WOB, Oscillation index.

**Table 2:** Kinematic characteristics (mean, range) of the three sperm subpopulations (SP1, SP2 and SP3) identified in sperm samples treated with 0, 0.05, 0.1 and 0.2 μM of YM-1, and stored at 17 °C for 21 days.

	SP1	SP2	SP3
N	53,590 (33.41%)	59,693 (34.21%)	47,133 (29.38%)
VCL (µM/s)	34.63 (10.00–107.04)	99.16 (51.30–280.04)	65.19 (10.04–165.52)
VSL (µM/s)	9.08 (0.00–43.04)	29.01 (0.00–131.44)	46.55 (7.80–143.82)
VAP (µM/s)	17.61 (2.68–79.22)	54.71 (7.07–136.11)	50.75 (8.72–139.79)
LIN (%)	27.56 (0.00–100.00)	28.87 (0.00–77.00)	71.97 (29.15–100.00)
STR (%)	53.12 (0.00–100.00)	52.56 (0.00–100.00)	90.47 (31.05–100.00)
WOB (%)	51.66 (7.33–100.00)	56.40 (6.75–97.93)	78.93 (31.94–100.00)
ALH (µm)	1.80 (0.31–4.62)	3.76 (1.34–10.92)	2.19 (0.22–5.12)
BCF (Hz)	4.57 (0.00–16.66)	12.04 (0.00–23.00)	9.43 (0.00–21.29)
DANCE (µM^2^/s)	72.14 (5.16–388.02)	391.65 (88.72–2498.66)	155.22 (4.34–741.71)
MDAabs	101.37 (0.00–218.65)	122.70 (20.67–253.75)	82.97 (0.00–273.87)

VCL, Curvilinear velocity; VAP, Average path velocity; ALH, Amplitude of lateral head displacement; BCF, Beat-cross frequency; VSL, Straight-line velocity; LIN, Linearity; STR, Straightness; WOB, Oscillation index.

**Figure 5:**
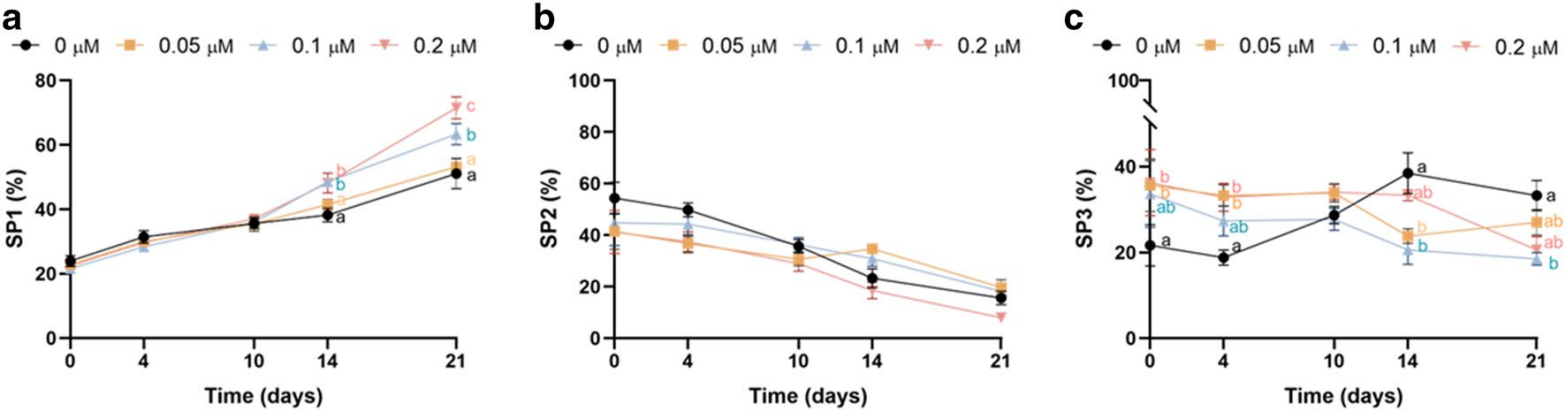
HSP70 activity inhibition during liquid storage of pig semen at 17 °C led to a redistribution of the percentages of sperm belonging to each of the three different subpopulations, based on their kinematic characteristics. Different letters indicate significant differences (*P* ≤ 0.05) between experimental groups (0, 0.05, 0.1 and 0.2 μM of YM-1) on a given time point (0, 4, 10, 14 and 21 days). Results are expressed as the mean ± SEM (N = 6).

The distribution of motile sperm in these subpopulations varied along the 21 days of experiment, as depicted in Fig. 5. Incubating sperm with the two highest concentrations of YM-1 significantly increased the percentages of SP1 on days 14 and 21. On the other hand, inhibition of HSP70 activity (0.05 and 0.2 µM of YM-1) significantly raised the percentages of SP3 on days 0 and 4. After long-term storage (days 14 and 21), the percentages of sperm belonging to SP3 decreased when samples were treated with 0.05 (day 14) and 0.1 µM of YM-1 (days 14 and 21).

### Inhibition of HSP70 activity has no effect on chromatin condensation

**Figure 6:**
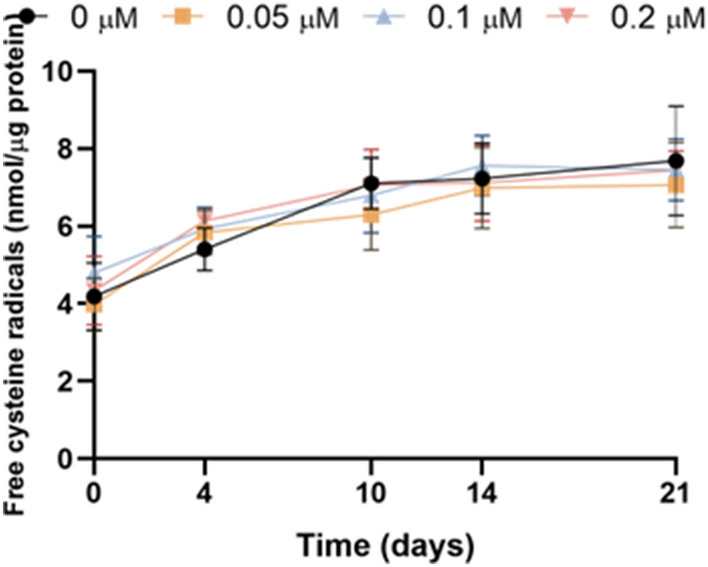
Inhibition of HSP70 activity with YM-1 during liquid storage of pig semen at 17 °C did not alter chromatin condensation, as evaluated with the levels of free cysteine radicals of sperm head proteins. Results are expressed as the mean ± SEM (N = 6).

Chromatin condensation, evaluated as free cysteine radicals of sperm head proteins, was not altered when HSP70 activity was inhibited with YM-1. The absence of any effect was consistent throughout the period of storage (Fig. 6).

## Discussion

Due to the limitations of freeze-thawing, liquid storage at 15–20 °C is the main method for pig sperm preservation^4,5^. While this temperature decreases cell metabolism, the extent of that reduction is lesser than that observed with lower temperatures (4–5 °C), which are used to preserve sperm in other species. In order to improve pig semen preservation technology and extend the storage period, light should be shed on the molecular mechanisms keeping the cell functional over liquid storage. As the preservation time is a limiting factor, better understanding what happens when sperm are stored at 15–20 °C may be of interest not only for pig industry but also for other farm animals. We, therefore, aimed to determine whether HSP70 is involved in the resilience of pig sperm to liquid storage. For this purpose, the activity of this protein was inhibited by different concentrations of YM-1 (0, 0.05, 0.1 and 0.2 µM) while semen was kept at 17 °C for 21 days. As previously described, HSP70 is associated with low sperm quality, and has been proven to play an anti-apoptotic role in somatic cells^14,16^.

We first confirmed that YM-1 can effectively inhibit the activity of HSP70 in pig sperm. This was consistent with previous observations in human somatic cells, where treatment with YM-1 also resulted in the inhibition of HSP70 activity^19–21^. In spite of this, HSP70 inhibition did not greatly affect sperm membrane integrity during liquid storage at 17 °C. As HSP70 is known to be an anti-apoptotic protein^22^, we rather expected to see a decrease in sperm viability when treating cells with YM-1. In fact, HSP70 is known to protect neural cells against apoptosis^21^, and its silencing triggers apoptosis in lung cells under in vitro hypoxia/reoxygenation conditions^23^. Our findings would also contrast with the fact that inhibition of HSP70 with YM-1 in HeLa cells induces their death^19^. The surprising sperm viability data were, however, consistent with those obtained from membrane lipid disorder and acrosome integrity tests. In effect, neither the lipid disorder of plasma membrane, which refers to the organization status of its phospholipids, nor acrosome integrity were impaired when HSP70 was inhibited with YM-1. These results suggest that, at least in pig sperm, HSP70 does not play a role in maintaining sperm membrane integrity. Nevertheless, and considering the disagreement with what observed in somatic cells, further research to elucidate whether inhibition of HSP70 during liquid storage could trigger apoptotic-like changes is warranted.

While, as aforementioned, inhibition of HSP70 with YM-1 did not affect the integrity of plasma and acrosome membranes of pig sperm stored at 17 °C, it did have a repercussion on different functionality parameters. In effect, we observed that the inhibition of this chaperone increased oxidative stress, as total ROS and superoxide levels were higher in sperm stored at 17 °C for a period equal to or longer than 10 days. As HSP70 is a molecular chaperone that maintains cell homeostasis by promoting proteostasis^24^, the inhibition of this protein could drive the accumulation of protein aggregates, which are a source of ROS^25^. This could explain why inhibiting HSP70 with YM-1 raises ROS levels in pig sperm, and would support that this protein is involved in keeping appropriate ROS levels in these cells. This rise in ROS levels would not be high enough to exert an obvious impact on plasma membrane integrity, which would not exclude the possibility of having other detrimental effects on sperm, as observed in goat and human sperm^26,27^.

We also found that inhibition of HSP70 with low concentrations of YM-1 increased Ca^2+^ levels and mitochondrial activity after 10 days of storage. Because mitochondria are one of the most important sources of ROS in mammalian sperm^28^, the increase observed in mitochondrial activity would match with that of ROS. All these findings concur that, during liquid storage at 17 °C, HSP70 activity contributes to keep mitochondrial function at a reduced level, as the inhibition of this chaperone with YM-1 produces the opposite effect. Since the main goal of liquid storage is to reduce mitochondrial activity as much as possible, as this allows for the prolongation of sperm lifespan^29^, we suggest that increasing HSP70 activity could have a positive effect. On the other hand, Ca^2+^ has an important role in triggering the intrinsic apoptotic pathway^30^, by causing the release of pro-apoptotic molecules from mitochondria^31^. In our work, however, the increase in intracellular Ca^2+^ levels was not accompanied by a decrease in sperm viability, as it would have been expected. These findings suggest a lack of relationship between the increase of Ca^2+^ levels modulated by HSP70 and the occurrence of apoptotic-like changes, notwithstanding this should be interrogated further.

Regarding sperm motility, inhibition of HSP70 increased progressive motility during the first days of storage at 17 °C as well as the percentage of sperm with high velocity, as discussed in the following paragraph. These data again support the fact that inhibition of HSP70 results in an increase of metabolic activity, which would agree with the previously mentioned effects on MMP, Ca^2+^ and ROS levels. It is widely known that mitochondrial activity is associated with sperm motility^32,33^, even though the ATP produced by mitochondria is not completely essential for sperm movement^34,35^. In spite of this, there was no obvious effect on total motility, as one would have reckoned^32^. Surprisingly, the effect of YM-1 on sperm motility was only observed on day 4. As the inhibitory effect on HSP70 activity was observed to be consistent throughout the entire storage period (i.e., 21 days), it would have been reasonable that the repercussion on sperm motility was kept over that time. Hence, and as described below, we ran another approach based on cluster analysis to better address the impact of the inhibition of HSP70 activity on sperm motility.

Although average values of kinematics parameters (e.g., VSL, VCL, VAP…) are used to illustrate further the characteristics of sperm movement^36^, this approach does not consider the great variability between sperm cells within a sample^37,38^. Thus, running cluster analysis on the basis of individual kinematic parameters allows for the establishment of sperm motile subpopulations, which has been revealed as a more powerful way to evaluate the nature of sperm motility^39^. In the present study, we identified three separate subpopulations based on sperm kinematics. These results agree with previous studies conducted on pig sperm that they also found three different motile subpopulations^40–42^. Our study detected a slow non-linear subpopulation (SP1), and two faster subpopulations with a similar VAP (SP2 and SP3), but with differences in linearity, as SP3 showed a more linear trajectory than SP2, which exhibited a higher VCL. Despite these findings differing from previous studies that identified a pattern of slow, intermediate, and fast motility for the three subpopulations^40,43^, the disparate experimental conditions could account for these differences. Interestingly, inhibition of HSP70 activity redistributed the percentages of sperm subpopulations after 14 and 21 days of liquid storage, with results differing from those observed during the first days of storage. While the percentages of sperm belonging to SP1 increased, the ones belonging to SP3 decreased. Our findings in sperm kinematic subpopulations agree with a previous study in cryopreserved bovine sperm, which showed that HSP70 levels were correlated with sperm motility, and reported that the loss of motility after freeze-thawing was concomitant with a decrease in HSP70 levels^44^. We thus suggest that HSP70 plays an important role in maintaining sperm motility during liquid storage, especially after 14 days. Surprisingly, and as mentioned before, we noticed that inhibition of HSP70 activity by YM-1 increased the percentages of SP3 after 0 and 4 days of storage. This biphasic effect, with the inhibition of HSP70 activity increasing the percentages of the fastest population (SP3) during the first days of liquid storage (days 0 and 4) and decreasing them at the end of the storage period (days 14 and 21), could be explained by a premature rise in sperm motility when HSP70 is blocked. Not only would this match with our findings in mitochondrial activity and ROS levels, but it would also suggest that sperm preservation is impaired when HSP70 activity is inhibited, because this results in an insufficient reduction of sperm metabolism^4^.

Finally, inhibition of HSP70 activity had no effect on chromatin condensation, which was evaluated through the levels of free cysteine radicals in sperm nucleoproteins. While no similar study has evaluated the effects of HSP70 inhibition on chromatin condensation, a previous work in human sperm reported that HSP70 expression was increased in infertile men with high sperm DNA fragmentation, assessed with the TUNEL assay^45^. Our results indicate that HSP70 is not involved in keeping chromatin condensed throughout liquid storage of semen at 17 °C, which, given that changes in chromatin condensation antecede those in DNA fragmentation^13^, suggests that it does play a major role in maintaining nuclear integrity.

In conclusion, the present study confirmed that YM-1 inhibits HSP70 activity in pig sperm. We also demonstrated that inhibition of HSP70 activity by YM-1 increases mitochondrial membrane potential during the first few days of storage, which is associated with a rise in progressive motility and in the percentage of sperm belonging to the fast and linear subpopulation. This could be regarded as a premature activation and could ultimately be detrimental to sperm preservation after 10 days of storage. In effect, inhibition of HSP70 had a negative impact at the end of the storage period, as not only did ROS and Ca^2+^ levels increase but also the percentages of SP1, which contained slow and non-linear motile sperm. All these findings support that HSP70 is involved in keeping sperm mitochondrial activity low, which positively contributes to their preservation in good fate at 17 °C.

## Materials and methods

### Samples

A total of 36 ejaculates were purchased from an artificial insemination center (Grup Gepork S.L., Masies de Roda, Spain), and used in the present study. As authors did not manipulate any animal for the purpose of this study but, instead, seminal samples were provided by the farm, the relevant Ethics Committee indicated that no ethical approval was required. Seminal doses came from Pietrain boars that were healthy and sexually mature, and were fed under controlled conditions. The farm followed the standard protocol for the production of seminal doses intended to AI. Ejaculates were collected using the hand-gloved method, diluted in a commercial extender (Vitasem LD; Magapor S.L., Zaragoza, Spain), and immediately transported to the laboratory at 17 °C. Six pools, each resulting from six ejaculates coming from separate animals, were used in the present study (N = 6). At the beginning of experiments, total motility ranged between 82 and 91%, progressive motility was 46–54%, and sperm viability varied between 89 and 92%. Each replicate was incubated with different concentrations (0, 0.05, 0.1 and 0.2 µM) of the HSP70 inhibitor YM-1, and stored at 17 °C for 21 days. On days 0, 4, 10, 14 and 21, sperm quality, functionality and free cysteine radicals were evaluated.

### Evaluation of HSP70 activity

Sperm samples were centrifuged at 600 g for 5 min, supernatants were discarded, and pellets were stored at − 80 °C until used. Before evaluation, pellets were thawed on ice and resuspended in 250 µL of a homogenization buffer [(20 mM Tris–HCl, 250 mM sucrose, 10 mM EGTA ethyleneglycol-bis(β-aminoethyl)-N,N,Nʹ,Nʹ-tetraacetoxymethyl ester (EGTA), 2 mM ethylenediaminetetraacetic acid (EDTA), 2 mM phenylmethanesulfonyl, 40 µg/mL leupeptin and 1 mM Na_3_VO_4_] at 4 °C. Following this, samples were centrifuged at 4 °C and 1000 g for 15 min. Supernatants were collected and split into two aliquots. The first one (10 µL) was intended to determine total protein content (Bio-Rad Protein Assay Dye), and the second was used to assess HSP70 activity with the Heat Shock Protein Assay Kit (Abcam, Cambridge, UK), following the manufacturer’s instructions. Two technical replicates were examined. Results were expressed as the ratio between micromoles of linked substrate per minute and milligrams of total protein.

### Flow cytometry

A CytoFLEX cytometer (Beckman Coulter, California, USA) was used to evaluate sperm quality and functionality through flow cytometry. To discriminate cells from debris, Forward Scatter (FSD) and Side Scatter Detectors (SSD) were utilized. In the present study, the following parameters were assessed: sperm viability (SYBR-14/propidium iodide [PI]), acrosome integrity (*Arachis hypogaea* (peanut) conjugated with FITC [PNA-FITC]/PI), membrane lipid disorder (merocyanine [M540]/Yo-Pro-1), intracellular superoxide levels (dihydroethidium [HE]/Yo-Pro-1), intracellular ROS levels (2′,7′-dicholorodihydrofluorescin [H_2_DCFDA]/PI), intracellular Ca^2+^ levels (Fluo3-AM/PI), and MMP (JC-1). Samples were excited through a blue laser (488 nm). The green fluorescence emitted by SYBR-14, Yo-Pro-1, PNA, JC-1 monomers, Fluo3 and DCF was detected with the FITC channel (525/40). On the other hand, the PE channel (585/42) was used to detect the orange fluorescence from JC-1 aggregates. Finally, the red fluorescence emitted by E was collected through the PE channel (585/42), while the fluorescence emitted by M540 was detected by the ECD channel (610/20), and that emitted by PI was collected through the PC5.5 channel (690/50).

In every analysis, samples were diluted in PBS to a final concentration of 4 × 10^6^ sperm/mL and subsequently stained with fluorochromes, as described below. Two technical replicates of at least 10,000 sperm each were analyzed for each sample and time point. Representative histograms of flow cytometry evaluations are provided in Supplementary Fig. 1.

### Sperm viability (SYBR-14/PI)

Sperm viability was determined following the protocol described by Garner and Johnson^46^. Briefly, samples were incubated with both SYBR-14 (final concentration: 7.6 µM) and PI (final concentration: 31.8 nM) at 38 °C in the dark for 10 min. As a result of this staining, four populations were identified: (i) viable green-stained sperm (SYBR-14^+^/PI^−^); (ii) non-viable red-stained sperm (SYBR14^−^/PI^+^); (iii) non-viable (moribund) green- and red-stained sperm (SYBR14^+^/PI^+^); and (iv) debris particles (SYBR-14^−^/PI^−^). Results were expressed as the recalculated percentage of viable sperm after excluding the percentage of debris particles.

### Acrosome integrity (PNA-FITC/PI)

Acrosome integrity was evaluated following the protocol described by Nagy et al.^47^. As indicated in this protocol, samples were co-stained with PNA-FITC (final concentration: 1.17 µM) and PI (final concentration: 5.6 µM) at 38 °C in the dark for 10 min. As sperm were not permeabilized, the following four populations were identified: (i) sperm with intact plasma membrane (PNA-FITC^−^/PI^−^); (ii) sperm with damaged plasma membrane and with an outer acrosome membrane that could not be fully intact (PNA-FITC^+^/PI^+^); (iii) sperm with damaged plasma membrane and without an outer acrosome membrane (PNA-FITC^−^/PI^+^); and (iv) sperm with damaged plasma membrane (PNA-FITC^+^/PI^−^). Hence, there were two main categories: (a) sperm with intact plasma membrane (PNA-FITC^−^/PI^−^), and (b) sperm having their plasma membrane and/or their outer acrosome membrane damaged (PNA-FITC^+^/PI^−^, PNA-FITC^+^/PI^+^, PNA-FITC^−^/PI^+^). Data were corrected excluding the percentage of debris particles, and the percentage of viable sperm with intact acrosome membrane (PNA-FITC^−^/PI^−^) was used to determine the acrosome integrity of samples.

### Membrane lipid disorder (M540/Yo-Pro-1)

To evaluate sperm membrane lipid disorder, samples were co-stained with M540 and Yo-Pro-1, following the protocol of Rathi et al.^48^ with some modifications^17^. Briefly, samples were stained with M540 (final concentration: 2.5 µM) and Yo-Pro-1 (final concentration: 25 nM) at 38 °C in the dark for 10 min. The basis of this protocol is that, when lipid disorder of plasma membrane is high, M540 intercalates and emits red fluorescence. Four populations were identified: (i) viable sperm with low membrane lipid disorder (M540^−^/Yo-Pro-1^−^); (ii) viable sperm with high membrane lipid disorder (M540^+^/Yo-Pro-1^−^); (iii) non-viable sperm with low membrane lipid disorder (M540^−^/Yo-Pro-1^+^); and (iv) non-viable sperm with high membrane lipid disorder (M540^+^/Yo-Pro-1^+^). Data were recalculated by excluding the percentage of debris, and the percentage of viable sperm with low membrane lipid disorder (M540^−^/Yo-Pro-1^−^) was considered further in statistical analyses.

### Intracellular superoxide levels (HE/Yo-Pro-1)

Co-staining with HE and Yo-Pro-1 was employed to determine intracellular superoxide levels, as described by Guthrie and Welch^49^. In the presence of superoxide, HE is oxidized into E^+^, which emits red fluorescence. The protocol consisted of incubation of samples with both HE (final concentration: 5 µM) and Yo-Pro-1 (final concentration: 31.25 nM) at 38 °C in the dark for 20 min. The E^+^ intensity in viable sperm (Yo-Pro-1^−^) was utilized to determine the intracellular superoxide levels in this population. Results were normalized against their respective control.

### Intracellular ROS levels (H_2_DCFDA/PI)

Intracellular ROS levels were assessed after co-staining with H_2_DCFDA and PI, following the protocol of Guthrie and Welch^49^. First, samples were incubated with H_2_DCFDA (final concentration: 100 µM) at 38 °C in the dark for 20 min. Subsequently, samples were stained with PI (final concentration: 12 µM) at 38 °C in the dark for 5 min. The DCF^+^ intensity in viable sperm (PI^−^) was used to determine total ROS levels. Data were normalized against the intensity of their respective control.

### Intracellular Ca^2+^ levels (Fluo3-AM/PI)

Sperm were co-stained with Fluo3-AM and PI to evaluate intracellular Ca^2+^ levels, following the protocol described by Harrison et al.^50^. Briefly, samples were stained with Fluo3-AM (final concentration: 1.2 µM) and PI (5.6 µM) at 38 °C in the dark for 10 min. The Fluo3^+^ intensity of viable sperm (PI^−^) was used to determine intracellular Ca^2+^ levels. Data were normalized against their respective control.

### Mitochondrial membrane potential (JC-1)

Mitochondrial membrane potential was determined by staining the samples with JC-1, following the protocol of Llavanera et al.^51^. Samples were incubated with JC-1 (final concentration: 750 nm) at 38 °C in the dark for 30 min. When samples have high MMP, JC-1 forms aggregates that emit red fluorescence, whereas when it is low JC-1 molecules remain as monomers, which emit green fluorescence. The ratio of fluorescence intensity between JC-1_agg_ and JC-1_mon_ was determined and used as a measure of MMP of samples.

### Evaluation of sperm motility

Sperm motility was assessed with a Computerized Sperm Class Analyzer (CASA) system, consisting of a phase contrast microscope (Olympus BX41; Olympus, Tokyo, Japan) connected to a computer equipped with ISAS software (Integrated Sperm Analysis System V1.0; Proiser SL, Valencia, Spain). Briefly, samples were pre-heated at 38 °C for 10 min, and 3 µL were subsequently loaded into a Leja chamber (Leja Products BV; Nieuw-Vannep, The Netherlands). A minimum of 1,000 sperm per sample were analyzed, and two technical replicates for each sample and time point were evaluated.

In all samples, percentages of total and progressively motile sperm were determined. Total motility was defined as the percentage of sperm with an average path velocity (VAP) higher than 10 µm/s, whereas progressively motile sperm were those with a straightness (STR) equal to or greater than 45%. For the study of motile sperm subpopulations, the following kinematic parameters were recorded for each spermatozoon: straight-line velocity (VSL, µm/s), curvilinear velocity (VCL, µm/s), VAP (µm/s), linearity index (LIN, %), STR (%), oscillation index (WOB, %), amplitude of lateral head displacement (ALH, µm), and beat-cross frequency (BCF, Hz). LIN was calculated as the ratio between VSL and VCL multiplied by 100; STR resulted from dividing VSL/VAP and multiplying by 100; and WOB was obtained by dividing VAP and VCL and multiplying by 100. The determination of the different sperm subpopulations is explained in the subsection about statistical analyses.

### Evaluation of chromatin condensation

Chromatin condensation was indirectly determined through the levels of free cysteine radicals of sperm head proteins. For this purpose, 5 mL of each sample were centrifuged at 600 g for 5 min. Supernatants were subsequently discarded, and pellets were resuspended in 1 mL of PBS. Thereafter, samples were again centrifuged under the same conditions, supernatants were discarded, and pellets were stored at − 80 °C until the day of analysis. After thawing, samples were resuspended in a cysteine buffer (50 mM Tris/HCl, 150 mM NaCl, 1% (v:v) Nonidet P-40, 0.5% (w:v) sodium deoxycholate, 1 mM Na_3_VO_4_, 10 µM/mL leupeptin, 1 mM benzamidine and 0.5 mM phenylmethyl sulfonyl; pH 7.4). Following this, samples were sonicated by applying 11 ultrasound pulses at maximum intensity, thus allowing for homogenization and detachment of heads from the tails. Free cysteine radicals in sperm head proteins were evaluated following the protocol of Brocklehurst et al.^52^, with some modifications^53^. Samples were first centrifuged at 850 g and 4 °C for 20 min, and supernatants and the upper layer of the pellet were discarded. Then, pellets were resuspended in 1 mL of cysteine buffer and, subsequently, 20 µL of the aliquot were mixed with 0.2 mM of 2,2′-dipyridil disulfide and incubated at 37 °C for 1 h. Finally, samples were evaluated with a spectrophotometer at a wavelength of 343 nm. Data corresponding to free cysteine radical levels of sperm head proteins were standardized against total protein content, which was determined with a commercial kit (Bio-Rad Protein Assay Dye) based on the Bradford method^54^. Two technical replicates were examined.

### Statistical analyses

A statistical package (SPSS Ver. 27.0 for Windows; IBM Corp., Armonk, NY, USA) was used to perform statistical analyses. First, data were tested for normality (Shapiro–Wilk test) and homoscedasticity (Levene test). The effects of inhibiting HSP70 with YM-1 on sperm quality and functionality were evaluated through a linear mixed model, and pair-wise comparisons were assessed by the post-hoc Bonferroni test. In the linear mixed model, the different time points were the intra-subjects factor, and the different treatments were the inter-subjects factor.

Kinematics-based sperm subpopulations were determined as described by Luna et al.^55^. Briefly, individual kinematic parameters were recorded for each spermatozoon, and utilized as independent variables in a Principal Component Analysis (PCA). The resulting matrix was rotated with the Varimax method, applying Kaiser normalization. Regression scores obtained were then used to perform a two-step cluster analysis based on the log-likelihood distance and the Schwarz’s Bayesian Criterion. Thanks to this approach, three motile subpopulations were identified, and each spermatozoon was automatically assigned to one of these subpopulations. Subsequently, the percentages of sperm belonging to each subpopulation were calculated for each treatment and time point, and compared through a linear mixed model (inter-subjects factor: treatment; intra-subjects factor: time of storage).

In all statistical analyses, the level of significance was set at *P* ≤ 0.05. Data were represented as mean ± standard error of the mean (SEM).

## Data Availability

The datasets used in the present study are available from the corresponding author on reasonable request.
